# Low-Molecular-Weight Secondary Metabolites from Fungi: *Cerrena unicolor* as a New Proposal of an Effective Preparation against *Rhabditis* Nematodes

**DOI:** 10.3390/molecules27051660

**Published:** 2022-03-03

**Authors:** Marta Ziaja-Sołtys, Przemysław Kołodziej, Dawid Stefaniuk, Anna Matuszewska, Magdalena Jaszek, Anna Bogucka-Kocka

**Affiliations:** 1 Chair and Department of Biology with Genetics, Medical University of Lublin, Witolda Chodźki Street 4A, 20-093 Lublin, Poland; przemyslaw.kolodziej@umlub.pl (P.K.); anna.bogucka-kocka@umlub.pl (A.B.-K.); 2 Department of Biochemistry, Maria Curie-Skłodowska University, Akademicka Street 19, 20-033 Lublin, Poland; dawid.stefaniuk@poczta.umcs.lublin.pl (D.S.); anna.matuszewska@poczta.umcs.lublin.pl (A.M.); magdalena.jaszek@poczta.umcs.lublin.pl (M.J.)

**Keywords:** fungi, low-molecular-weight metabolites, *Nematodes*, antiparasitic properties

## Abstract

Plants and fungi are known as a valuable source of natural medicines used in the treatment of various diseases. Many of them are used to treat human and animal gastrointestinal diseases caused by parasites. The aim of this study was to investigate for the first time the antinematode properties of extracellular low-molecular subfractions (ex-LMS) obtained from the liquid growth medium of idiophasic *Cerrena unicolor* cultures. The fungal fractions were isolated according to a procedure previously described by Jaszek et al. The in vitro tests were performed using nematodes of the *Rhabditis* genus. As demonstrated by the results, the total fraction with a molecular weight < 10 kDa (CU-A) and the 0.02–1.5 kDa fraction (CU-B) had nematicidal activity. It was found that the analyzed substances induced movement disturbances caused by the paralysis of the back part of the nematode’s body. The degree of body paralysis was proportional to the increase in the concentration of the tested fractions. Summarizing the obtained results in the context of the available literature data, it seems that *C. unicolor* may be a good new candidate for research on nematode infections.

## 1. Introduction

Nematodes are present in up to 80% of all known animals. They are present all over the world. Many species of unsegmented roundworms of the phylum *Nematoda* are harmful because they parasitize on humans, animals, plants, and fungi. Gastrointestinal infections in humans and animals caused by parasitic nematodes is a serious health and economic problem affecting both poor, developing and rich countries [1,2].

According to a report by the World Health Organization (WHO), about 1.5 billion people in the world were infected with intestinal nematodes in 2018. Internal infection with parasitic nematodes significantly interferes with the proper functioning of the human body, and the effects of the disease are persistent and particularly dangerous in children [3]. The high incidence in and mortality of livestock and wild animals caused by helminth infections result in huge global economic losses in the production of food and products of animal origin [4,5]. Sheep haemonchosis is a disease caused by *Haemonchus contortus* nematodes of the Trichostrongylidae family. The disease causes anemia, diarrhea, weight loss, low production performance, and even the death of young animals, thereby causing great economic losses to breeders [6,7].

The pathogenesis of parasitic infections varies depending on the species of nematodes that causes the disease. In addition, there are simultaneous infections with several species of worms, which deepens the harmful effect on the host organism [8,9].

The currently used anthelmintic drugs include, but are not limited to, aminoacetonitrile derivatives (e.g., monepantel), aminophenylamidine (e.g., tribendimidine), benzimidazoles (e.g., albendazole and mebendazole), macrocyclic lactones (e.g., ivermectin), spiroindoles (e.g., derquantel), and tetrahydropyrimidines (e.g., pyrantel). The mechanisms of action of these substances differ. Albendazole, which was used in our experiments as a positive control, exerts its antiparasitic effect by the inhibition of tubulin polymerization, including the absorption of glucose by the parasite, thereby causing its death. It is active against eggs, larvae, and adults of intestinal parasites [4,10,11].

The ease of administration and relatively low purchase costs of commercially available antiparasitic drugs have led to their excessive use, especially in animal breeding, resulting in increased resistance of nematodes to these substances. The presence of residues of anthelmintic substances in food of animal origin and in the environment is also a problem. Their deleterious and toxic effects on human health have been reported [1,4]. 

Therefore, there is an urgent need to look for new, effective, natural anthelmintic substances that are safe for humans and animals and less detrimental to the environment.

Plants and fungi are a valuable source of natural medicines that have been used for the treatment of various diseases for years. The antibacterial, antiviral, antioxidant, or antitumor effects of plant and fungal substances are widely known. Many of them are also used to treat human and animal gastrointestinal diseases caused by parasites [1,12,13,14,15,16].

One of the best-known medicinal mushrooms is *Ganoderma* sp., producing substances of varying chemical nature with many proven biological properties, e.g., anticancer, immunomodulatory, antihypertensive, antiandrogenic, and antiparasitic [17,18].

Secondary metabolites of fungi or plants are natural sources of substances with biological activities; they are also important for the development of new, more perfect drugs. As suggested by Patel [19], many species of fungi, especially belonging to the genera of the class Basidiomycetes, can be treated as miniature factories of a huge number of substances with potent biological properties. Bioactive substances isolated from fungi can be divided according to the size of their molecules into substances with high and low molecular weight. The first group includes mainly polysaccharides and enzymes, while the low-molecular-weight compounds are represented by, e.g., terpenoids, phenols, and indoles [20,21,22]. Many plant-derived products from the group of alkaloids, terpenes (e.g., artemisinin), and phenols have shown effective selective antiparasitic properties. However, they have mainly been tested against parasitic protozoa [23].

Many active substances have also been isolated from wood degrading mushroom species. One of the efficient sources of multiple bioactive compounds is the white rot fungus *C. unicolor* (Table 1) [12,15,24]. During their growth, white rot fungi produce large amounts of side products during the synthesis of enzymes, for instance, laccase, in the liquid culture medium. The antiviral, antibacterial, pro- and antioxidant, anticancer, or immunomodulatory activity of substances derived from *C. unicolor* has been demonstrated in recent years [25]. Fractions of extracellular low-molecular-weight secondary metabolites isolated from idiophasic cultures of *C. unicolor* were found to modulate selected parameters related to hemostasis processes and antiproliferative, proapoptotic, and migration-inhibiting properties. The acceleration of fibrin clot formation in the presence of fibrinogen and thrombin as well as an enhanced fibrin cross-linking degree were observed [15,24,26].

Some of the above-mentioned properties can be related both to the presence of bioactive peptides, polysaccharides, and phenolic compounds and to the high antioxidant potential of the fractions tested. In our previous research, we found that the highest concentration of low-molecular-weight peptides, determined with the Bradford method, was recorded for fraction 6. It also had the highest concentration of phenolic compounds determined by the diazosulfanilamide method as well as the highest potential for scavenging stable free radicals 2,2’-azino-bis(3-ethylbenzothiazoline-6-sulfonic acid) (ABTS) and 2,2-diphenyl-1-picrylhydrazy (DPPH), compared to the standard antioxidant-Trolox (Table 1). More detailed information on the analytical methods used and the results obtained can be found in reference [25].

*Rhabditis* nematodes were used as a research model. The nematode *Caenorhabditis elegans*, a well-studied model organism, belongs to the same group. These nematodes live naturally in the soil and on decaying plant remains, fruit, and fungi rich in microorganisms [27]. *Rhabditis* nematodes can cause infections of the external auditory canal, the gastrointestinal tract, and the urinary tract in humans [28,29,30]. The research model used in the study makes it possible to evaluate the antinematode properties of substances on the basis of observation of the behavior, movement, and reproduction of nematodes exposed to these agents. If the tested substances are effective against *Rhabditis* in laboratory cultures at low concentrations, it can be assumed that they may also be active against other related nematodes [31]. It is also important that the discussed research model enables the assessment of the properties of the tested substances against adult nematodes, which are the most harmful to humans and animals as definitive hosts. Moreover, the *Rhabditis* used for the research does not require large financial outlays. To the best of our knowledge, in the literature available in the databases of scientific journals, there are no research results on the antiparasitic properties of extracts from *C. unicolor*.

The aim of this work was to investigate for the first time the antinematode properties of extracellular low-molecular-weight subfractions (ex-LMS) obtained from the liquid growth medium of idiophasic *C. unicolor* cultures. 

## 2. Results

**Figure 1:**
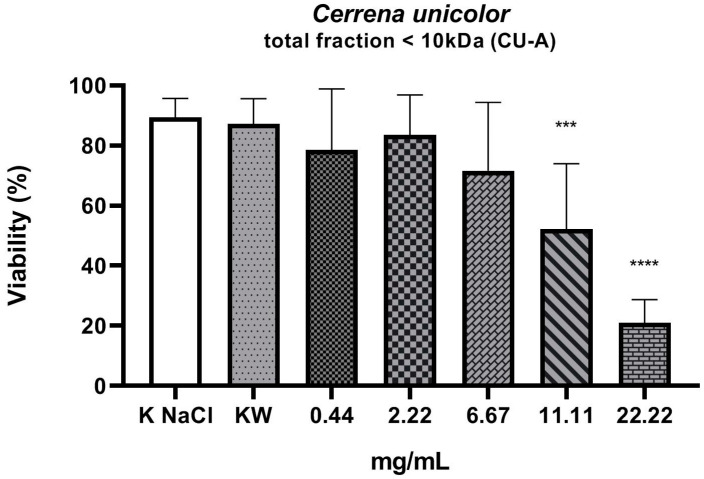
Viability (%) of *Rhabditis* sp. nematodes after 24 h of exposure in five experimental concentrations of the total < 10 kDa fraction tested (CU-A). KNaCl, negative control; KW, solvent control (*p* < 0.0001 ****, 0.0001 to 0.001 ***); ┬, means (standard deviation (SD)).

**Figure 2:**
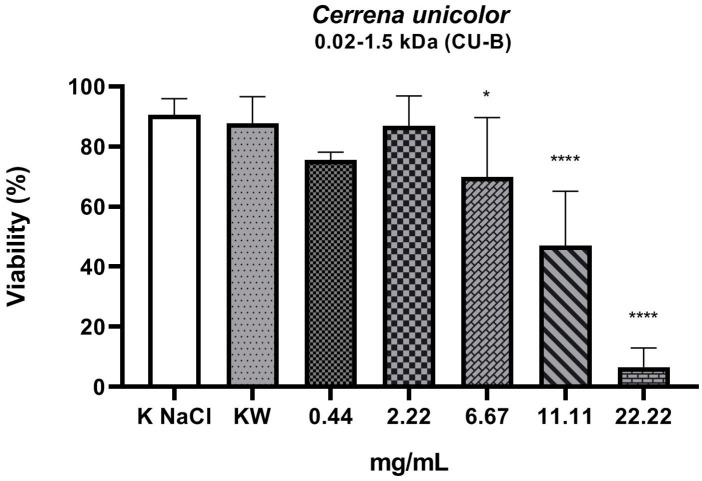
Viability (%) of *Rhabditis* sp. nematodes after 24 h of exposure in five experimental concentrations of the 0.02–1.5 kDa fraction (CU-B) tested. KNaCl, negative control; KW, solvent control (*p* < 0.0001 ****, 0.01 to 0.05 *); ┬, means (standard deviation (SD)).

**Figure 3:**
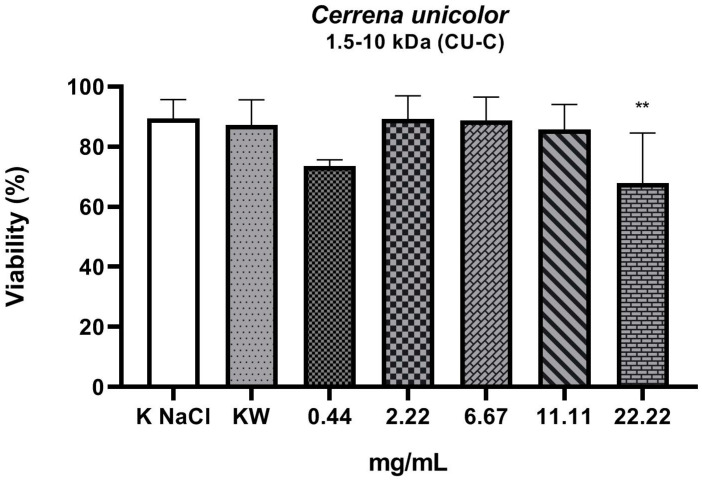
Viability (%) of *Rhabditis* sp. nematodes after 24 h of exposure in five experimental concentrations of the 1.5–10 kDa fraction tested (CU-C). KNaCl, negative control; KW, solvent control (*p* = 0.001 to 0.01 **); ┬, means (standard deviation (SD)).

**Figure 4:**
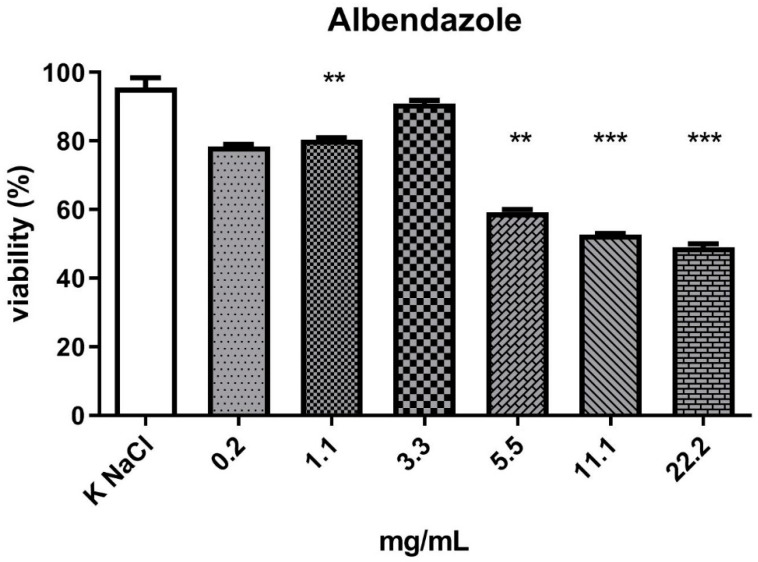
Viability (%) of *Rhabditis* sp. nematodes after 24 h of exposure in six experimental concentrations of albendazole. KNaCl, negative control; (*p* < 0.0001 to 0.001 ***, 0.001 to 0.01 **); ┬, means (standard deviation (SD)).

The experiment investigated the nematicidal activity of three fractions isolated from liquid culture of the *C. unicolor* wood rot fungus. From the total fraction containing all compounds weighing less than 10 kDa (CU-A), fractions of compounds with weight in the range of 0.02–1.5 kDa (CU-B) and 1.5–10 kDa (CU-C) were isolated. As shown by the results, the total fraction (molecular weight < 10 kDa) (Figure 1) and the 0.02–1.5 kDa fraction (Figure 2) showed nematicidal activity. The concentration values of both fractions, which resulted in a 50% reduction in the number of viable nematodes in the tested sample, were 11.7 mg/mL (fraction < 10 kDa) and 9.4 mg/mL (0.02–1.5 kDa), respectively. The highest concentration, of 22.22 mg/mL, of both these fractions reduced the viability of the nematodes to 21% and 13.8%, respectively. The part of the total fraction containing compounds with a mass in the range of 1.5–10 kDa turned out to have a low nematicidal potential (Figure 3), reducing the viability of *Rhabditis* sp. to 68% at a concentration of 22.22 mg/mL, compared to the control sample (89%). However, this decrease was statistically significant. The tested fractions of *C. unicolor* showed stronger nematicidal properties at lower concentrations than the drug albendazole used in the treatment (Figure 4).

**Figure 5:**
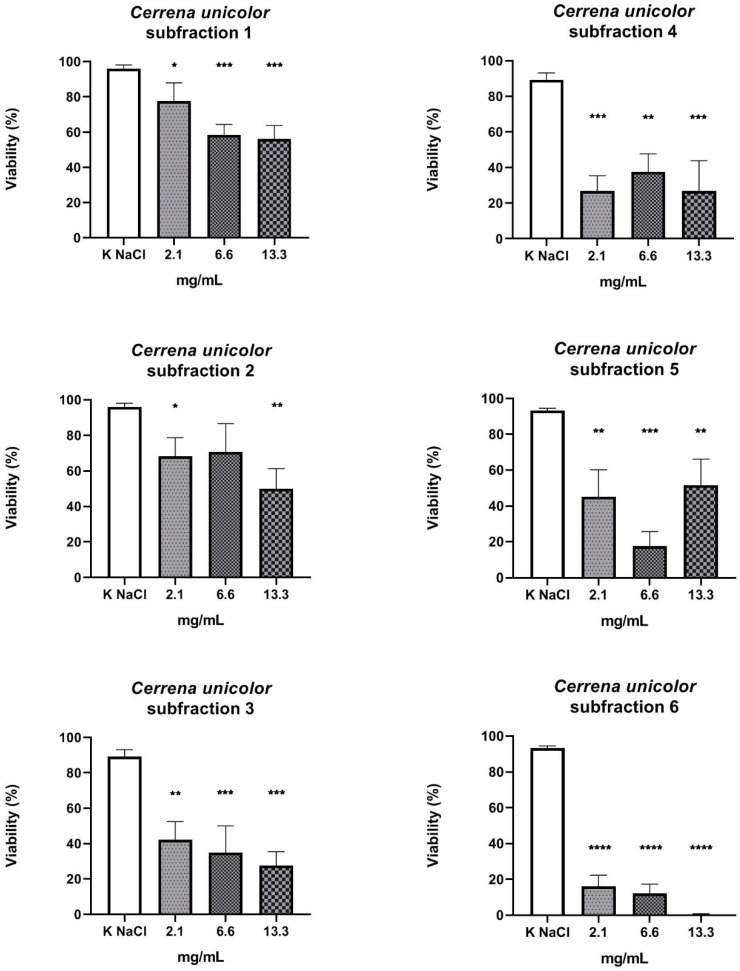
Viability (%) of *Rhabditis* sp. nematodes after 24 h of exposure in three experimental concentrations of the tested subfractions S1-6 of 0.02–1.5 mg/mL (CU-B) fraction. KNaCl, negative and solvent control; (*p* < 0.0001 ****, 0.0001 to 0.001 ***, 0.001 to 0.01 **, 0.01 to 0.05 *); ┬, means (standard deviation (SD)).

The next step in the research was to determine the antinematode properties of six subfractions isolated from the 0.02–1.5 kDa fraction. All subfractions reduced the viability of nematodes grown in their presence. However, the strongest nematicidal properties were observed after the application of subfraction 6, as the concentrations of 2.1 mg/mL and 6.6 mg/mL reduced the viability of the nematodes to 17.27% and to 13%, respectively, and the highest concentration (13.3 mg/mL) showed killing activity against all treated nematodes (Figure 5).

**Figure 6:**
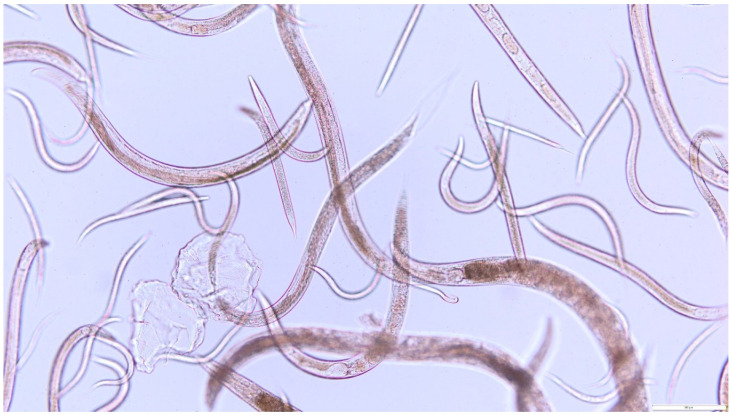
*Rhabditis* sp. nematodes after 24 h cultivation against 0.6% NaCl (control culture).

**Figure 7:**
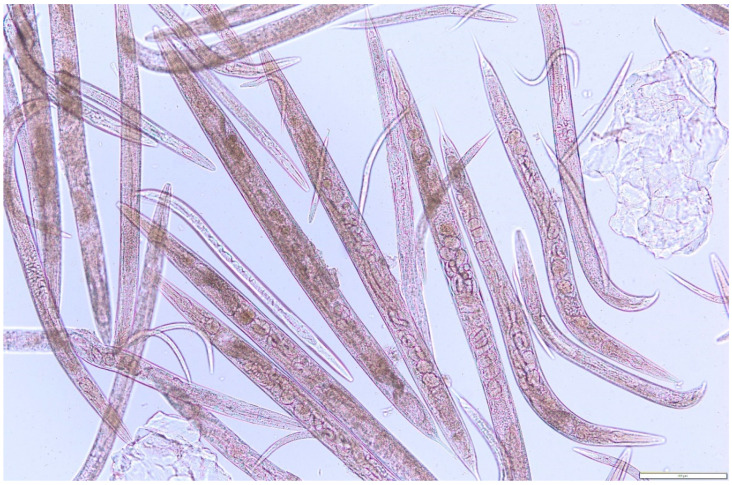
*Rhabditis* sp. nematodes after 24 h cultivation against the low-molecular-weight fraction of 0.02–1.5 kDa (CU-B) isolated from the liquid culture of *Cerrena unicolor* fungus.

Deformation and changes in nematode motility were assessed as well. The tested substances were found to cause movement disorders due to paralysis of the posterior part of the nematode’s body. The degree of body paralysis was proportional to the increase in the concentration of the tested fractions. Nematodes treated for 24 h with the highest dose (22.22 mg/mL) of the 0.02–1.5 kDa fraction and (13.3 mg/mL) of subfraction 6 either died or were almost completely paralyzed. The tested substances had a visible negative effect on both adult and larval forms of the nematodes (Figure 6 and Figure 7).

## 3. Discussion

Diseases caused by nematodes constitute the vast majority of parasitic diseases in humans. In this context, the increasing drug resistance in parasites is an extremely important problem. There is a great need to search for natural and synthetic compounds acting against nematodes and exhibiting effectiveness, safety for humans, and low environmental toxicity. In recent years, special attention has been paid to fungi as a source of secondary metabolites with a wide range of biological activities. Some fungal species produce compounds and hydrolases causing structural destabilization of the epidermis of nematodes, thereby leading to their death. These compounds and enzymes are active against both free-living and intestinal parasitic nematodes [32].

Mushrooms are traditionally used in many different cultures as food or various supplements to maintain health and to prevent and treat various diseases. They have been found to exhibit over 100 therapeutic properties, including antitumor, immunomodulatory, antioxidant, radical-scavenging, cardiovascular, antihypercholesterolemic, antiviral, antibacterial, antiparasitic, antifungal, detoxifying, hepatoprotective, and antidiabetic effects [33,34,35,36]. They are an excellent source of dietary supplements. The production of such supplements has many advantages because the vast majority of mushrooms used in this process are commercially grown but not harvested from the natural environment. Furthermore, these mushrooms easily reproduce vegetatively and retain the properties of one clone. In addition, their mycelium can be stored for a long time, which does not affect the results of genetic and biochemical tests. A great advantage is that many fungi can be grown as mycelial biomass in liquid cultures [37]. Basidiomycetes contain secondary metabolites in fruit bodies, cultured mycelium, and cultured broth [38]. *Rhabditis* nematodes are the main model organism used for a wide variety of physiological processes due to such advantages as the small body size; short lifespan; ease of maintenance; and, first of all, physiological similarity to mammals [39].

Edible fungi of the genus *Pleurotus* have been shown to have various therapeutic properties, including nematicidal activity. Chromatographic fractionation of the hydroalcoholic extract from *P. djamor* fruiting bodies was performed, and metabolites with nematicidal activity were identified. The tests were carried out in vitro against *H. contortus* and in vivo against gerbils artificially infected with *H. contortus*. The results showed that the tested fraction at concentrations from 5 mg/mL caused 100% inhibition of hatching from eggs. They also showed over 97.2% of larvicidal activity after 2 h at concentrations up to 20 mg/mL. The in vivo evaluation of the fractions revealed a 92.56% reduction in the number of *H. contortus* larvae. The compounds identified in this fraction were a 9:1 mixture of allitol and an unidentified terpene. It was found that the tested fraction can be used to combat *H. contortus* infection [40]. 

We assessed the viability of *Rhabditis* after 24 h of exposure to substances derived from low-molecular-weight fractions isolated from the *C. unicolor* fungus. The number of live and dead individuals was assessed. The nematodes were also observed under stereoscopic and light microscopes in order to assess their behavioral characteristics. Certainly, in future studies, we will try to elucidate the mechanism of this process and make attempts to isolate specific, homogeneous, active compounds and determine their chemical structure by means of LC-MS and NMR analyses. In our experiment, the negative control (0.6% NaCl) showed that 11% of the nematodes were dead. This result is related to the fact that, in a liquid culture, the nematodes were washed out of the agar culture, where a small percentage of the nematodes are dead in standard conditions. Analysis of the results of the 24 h *Rhabditis* culture against the CU-A and CU-B fractions at a concentration of 0.44 mg/mL showed a slightly greater decrease in the number of viable nematodes than at a concentration of 2.22 mg/mL. As shown by the statistical analysis, this value is not statistically significant. In the case of the CU-C fraction at the concentration of 0.44 mg/mL, a greater decrease in nematode viability was observed than in the case of the three subsequent concentrations (2.22, 6.67, and 11.11 mg/mL). However, the differences between these values were also not statistically significant.

In many studies, a deviation from the distribution of data resulting from the viability analysis (nematodes, cells) is observed against increasing concentrations of test substances. It concerns the low concentration (sub-threshold dose) of the test substance. In the presence of such a dose, a greater decrease in viability is observed than at slightly higher doses of the substance. This is followed by a definite decrease in viability after high doses are applied. In the case of our experiment, a similar phenomenon was observed not only in the test samples but also after incubation of nematodes in the presence of increasing concentrations of albendazole (positive control). The mechanism of this phenomenon is not explained. These results were repeated in subsequent replications; therefore, a more detailed analysis of fractions and subfractions and a study of the mechanism of their action on the organism of *Rhabditis* nematodes may explain these results in detail. 

An important aspect of using *Rhabditis* as a model for the study of anthelmintics is the comparative physiology and pharmacology of Nematoda. These similarities are important because most anthelmintics act on the neuromuscular system and the key transduction molecules in the nervous system are highly conserved from worm to human [11,41]. As a result of nematode culturing in the presence of the increasing concentrations of the tested CU-A, CU-B, and CU-C fractions, a progressive reduction in motility and subsequent death of the nematodes was observed. The ability of the nematodes to move as a result of the action of the fractions decreased with the increasing fraction concentration. Similar results were obtained for *Rhabditis* cultures compared to the positive control albendazole. Interestingly, the highest concentration, of 22.2 mg/mL, of the CU-B fraction (0.02–1.5 kDa) exerted a stronger nematicidal effect than the same concentration of albendazole. The reason for this phenomenon may be the synergistic effect of the constituent fractions (CU-B). As we do not yet know the mechanism of action of the CU-B fraction, we cannot compare it to the mechanism of action of albendazole. We can assume that they may be different. The anthelmintic efficacy of benzimidazoles is due to their ability to selectively interact with β-tubulin, resulting in disturbances in movement, reproduction, and damage to oocytes, as these processes require microtubule integrity [11]. 

Some authors have reported the likely mechanisms of action of bioactive compounds against parasite survival. According to literature reports, some alkaloids inhibit the synthesis of DNA, RNA, and lipids [42]. Free fatty acids (C14, C16, C18) have been reported to dissolve the cell membrane, interfere with nutrient absorption, inhibit enzyme binding, and generate peroxidation products that become toxic to microorganisms [43]. Other studies have demonstrated that flavonoids can form complexes with extracellular and soluble proteins bound to the cell membrane, which can cause changes in membrane function, and that phenolic acids and flavonoids derived from fungi may inhibit the activity of protein kinase [44]. 

Zhao et al. [45] obtained results indicating the lethal potential of the lectin from *G. lucidum* against plant parasitic nematodes *Heterodera glycines* and *Ditylencheus dispaci*, but it was not strong enough to be commercially applicable. 

Lectin identified in the fruiting bodies of the clouded agaric *Clitocybe nebularis* (CNL) is a small and stable protein exhibiting several glycan-binding specificities. CNL forms homodimers, which is essential for its functionality. This protein specifically binds GalNAc terminal residues and glycan epitopes containing the human blood group A determinant as well as less frequently other β-galactosides. The presence of specific target glycans on nematodes and cancer cells determines the nematotoxic and cytotoxic effects of CNL on leukemic T lymphocytes [46]. The extracts of the fruiting body and mycelium of *Agaricus blazei* are rich in phenolic compounds and have shown anthelmintic effects [47]. Based on their positive anthelmintic activity, five diastereomeric polyketide glycosides, roselipins, were isolated from the acetone extract of *Clonostachys candelabrum*. Linoleic acid and aurantiogliocladin were also isolated, but their activity was much weaker than that of roselipins [48]. 

So far, most studies on the activity of antiparasitic extracts and secondary metabolites isolated from higher fungi have been performed in vitro. Future research should involve more fungal species and evaluate their antiparasitic activity, mechanism of action, and identification of lead compounds so that they can be used for the effective and safe development of antiparasitic drugs [49].

## 4. Materials and Methods

### 4.1. Strain, Medium, Growth Processing

The *Cerrena unicolor* (Bull.ex Fr.) Murr strain was obtained from the culture collection of the Regensburg University and deposited as strain number 139 in the fungal collection of the Department of Biochemistry, Maria Skłodowska-Curie University, in Poland (ITS sequence deposited in GenBank under accession number DQ056858). The process of fungal culturing was conducted according to the procedure described earlier by Jaszek et al. [24] and Matuszewska et al. [25]. Briefly, the submerged cultivation was performed in an air-lift bioreactor (fermentor) containing 8 L of sterilized and optimized Lindenberg and Holm medium (composed of glucose 10 g/L, L-asparagine 1.5 g/L, MgSO_4_ × 7 H_2_O 0.5 g/L, KH_2_PO_4_ 0.47g/L, Na_2_HPO_4_ × 12 H_2_O 0.48 g/L, yeast extract 0.1 g/L, Mn(CH_3_COO)_2_ × 4 H_2_O 12 mg/L, Zn(NO_3_)_2_ × 6 H_2_O 3.14 mg/L, CuSO_4_ × 5 H_2_O 3.9 mg/L, Ca(NO_3_)_2_ × 4 H_2_O 50 mg/L, FeCl_3_ × 6 H_2_O 3.2 mg/L, thiamine 50 μg/L, final pH 5.5) at 26 °C [50]. The fermentor was inoculated with homogenized 10-day-old stationary Erlenmayer flask cultures grown on 100 mL of the Lindenberg and Holm medium (10% of total volume). The culture was aerated at the level of 1 L/min. The incubation lasted until the idiophase began, which was determined according to Jennings and Lysek’s recommendation; in brief, the glucose (main carbon source) consumption curve and the mycelium dry mass increase curve were determined. The moment of intersection of these two curves determines the transition of fungal cultures from the trophophase (logarithmic growth of the mycelium biomass) to the idiophase (a phase in which the production of secondary metabolites dominates) [51].

### 4.2. Preparation of Fungal Fractions

The process of isolation and preparation of the three studied fungal fractions and their characteristics are presented in detail by Jaszek et al. [24] and Matuszewska et al. [25]. Briefly, 10-day-old cultures in the idiophase were harvested and filtered through Miracloth (Calbiochem). After separation of the mycelium, the liquid culture medium was centrifuged at 10.000× *g* for 15 min and next divided into two fractions on the ultrafiltration system Prep/Scale-TFF (Millipore, Bedford, MA, USA) with a 10 kDa cut-off. The obtained permeate was the total, extracellular, low-molecular-weight (<10 kDa) fraction (CU-A). The ex-LMS (CU-A) was further processed using reverse osmosis membrane TFC-75F (Aquafilter Inc., Hunt Valley, MD, USA) to increase the concentration and fractionated on a Sephadex G-15 column (25 × 5 cm) into two subfractions, 1.5–10 kDa (CU-C) and 0.02–1.5 kDa (CU-B). The resulting preparations where freeze-dried. The fraction corresponding to a mass below 1.5 kDa (CU-B) was further fractionated on a 2.5 × 120 cm Sephadex G-15 column. The resulting six subfractions (S1, S2, S3, S4, S5, and S6) were freeze-dried and analyzed [24]. The obtained fractions were dissolved in distilled water and diluted to the studied concentrations.

### 4.3. Nematicidal Activity

*Rhabditis* sp. free-living nematodes in the soil in the natural environment were used in the research. The culturing of nematodes and the research methodology for the assessment of nematode properties were developed by the Chair and Department of Biology and Genetics of the Medical University of Lublin, patent no. 232918 (Bogucka-Kocka and Kołodziej). The *Rhabditis* sp. nematodes come from the collection of the Department of Biology and Genetics of the Medical University of Lublin.

In vitro nematode cultures were established in 6-well sterile plastic plates on a solid agar medium supplemented with fetal bovine serum and grown for 5 days at room temperature (21 °C) to obtain all nematode growth forms. The nematodes were then eluted from the solid agar medium with 0.6% NaCl solution and transferred to new sterile 24-well plates with liquid medium (0.6% NaCl) in a volume of 400 μL per well. The initial solutions of the total <10 kDa fraction (CU-A), the 0.02–1.5 kDa fraction (CU-B), and the 1.5–10 kDa fraction (CU-C) were prepared at the concentration of 200 mg/mL. Then, the fractions tested in the liquid medium were added (50 μL) to the cultures at the experimentally selected concentrations ranging from 0.44 to 22.2 mg/mL. The initial concentration of the S1–S6 subfractions was 120 mg/mL. Then, the tested S1–S6 subfractions were added to the cultures at the experimentally selected concentrations ranging from 2.1 to 13.3 mg/mL. Dilutions of the tested fractions were made in deionized water. Positive control cultures were also performed with the commercially available antiparasitic drug albendazole (a concentration range of 0.2–22.2 mg/mL was used) and two negative control cultures with 0.6% NaCl and water as the solvent of the tested fractions, respectively. The albendazole concentrations were experimentally selected so that their range was similar to the concentrations of the fractions tested. After 24 h exposure at room temperature (21 °C), the nematodes were observed under optical and stereoscopic microscopes. The viability, deformities, and disorders of their development were assessed. The viability of larvae and adults was assessed on the basis of their behavioral characteristics, i.e., mobility, reproduction, and the proportion of adults to larvae.

The live and dead individuals in the liquid culture were counted on microscope slides. In addition, objective evaluation of live and dead individuals was carried out on the basis of observations after staining the nematodes with methylene blue. Dead nematodes become stained, while live nematodes do not. In each observed preparation, live and dead individuals were counted and their total number was 100%. All controls were treated identically to the test samples. The experiment was performed in three independent replications [52].

### 4.4. Statistical Analysis

Statistical analysis was performed using the GraphPad Prism 8.2.0 software (GraphPad Software, San Diego, CA, USA) for a one-way analysis of variance (ANOVA), where statistically significant differences were adopted with the coefficient value of *p* < 0.0001 ****, 0.0001 to 0.001 ***, 0.001 to 0.01 **, 0.01 to 0.05 *, and ≥0.05 not significant.

## 5. Conclusions

Summarizing the obtained results in the context of the available literature data, it seems that *C. unicolor* may be a good new candidate for research related to nematode infections. The possibility of obtaining an effective preparation containing bioactive low-molecular-weight secondary metabolites in standardized conditions is additionally important from the point of view of medical biotechnology.

## 6. Patents

On the basis of the research results presented in the work, a patent was granted for the invention by the Patent Office of the Republic of Poland (No. 237995). 

## Figures and Tables

**Table 1:** Biochemical parameters (concentrations of protein, phenolic compounds, and total sugars) and antioxidant properties of the 1.5–10 kDa (CU-C) and 0.02–1.5 kDa (CU-B) fractions and CU-B subfractions S1–S6 from *Cerrena unicolor*; the values are presented as the mean of triplicates with a standard deviation (±).

	Protein (μg/mL)	Total Sugars (μg/mL)	Phenolic Compounds (μg/mL)	Antioxidant Properties (mM Trolox/g)		
				ABTS	DPPH	
**1.5–10 kDa (CU-C)**	4.3 ± 0.3	329.0 ± 38.6	64.9 ± 0.9	250.2 ± 0.5	52.0 ± 0.7	Unpublished data
**0.02–1.5 kDa (CU-B)**	10.9 ± 1.8	220.2 ± 2.8	55.4 ± 1.3	176.2 ± 0.4	24.8 ± 0.9	Matuszewska, 2019 [25]
**S1**	8.0 ± 0.4	253.8 ± 31.4	64.7 ± 0.2	308.8 ± 1.1	117.7 ± 7.2	
**S2**	1.9 ± 0.1	287.2 ± 44.1	50.5 ± 1.5	243.1 ± 0.1	119.5 ± 5.3	
**S3**	0 ± 0.0	215.5 ± 11.1	57.9 ± 1.2	169.0 ± 0.1	0.0 ± 0.2	
**S4**	2.3 ± 0.1	404.4 ± 30.5	61.5 ± 2.1	239.3 ± 1.0	91.5 ± 5.1	
**S5**	3.7 ± 0.2	328.7 ± 33.6	57.8 ± 1.1	303.0 ± 0.6	119.6 ± 4.3	
**S6**	31.8 ± 0.5	190.4 ± 44.2	73.5 ± 1.1	569.8 ± 3.8	168.4 ± 3.2	

## Data Availability

Not applicable.
